# Supplementary material for: Personalized risk stratification in colorectal cancer via PIANOS system
Source: Nat Commun. 2025 Jul 16;16:6561. doi: 10.1038/s41467-025-61713-1 (PMC12267411; doi:10.1038/s41467-025-61713-1)
Supplement: Supplementary file 1 — Supplementary Information [file 41467_2025_61713_MOESM1_ESM.pdf]

Personalized Risk Stratification in Colorectal Cancer via PIANOS System

Du Cai[1,2,3,#], Haoning Qi[1,2,3,#], Qiuxia Yang[4,#], Huayu Li[5,#], Chenghang Li[6], Chuling Hu[1,2,3], Baowen Gai[1,2,3], Xu Zhang[7], Yize Mao[8,*], Feng Gao[1,2,3,*] , Xiaojian Wu[1,2,3,*]

1.Department of General Surgery (Colorectal Surgery), The Sixth Affiliated Hospital, Sun Yat-sen University, Guangzhou, P. R. China

2. Guangdong Provincial Key Laboratory of Colorectal and Pelvic Floor Diseases, The Sixth Affiliated Hospital, Sun Yat-sen University, Guangzhou, China

3. Biomedical Innovation Center, The Sixth Affiliated Hospital, Sun Yat-sen University, Guangzhou, P. R. China

4. Department of Radiology, State Key Laboratory of Oncology in South China, Guangdong Provincial Clinical Research Center for Cancer, Sun Yat-sen University Cancer Center, Guangzhou 510060, P. R. China

5. State Key Laboratory of Oncology in South China, Guangdong Provincial Clinical Research Center for Cancer, Sun Yat-sen University Cancer Center, Guangzhou 510060, P. R. China

6. Artificial Intelligence Thrust, The Hong Kong University of Science and Technology, Guangzhou, China

7. Center for Reproductive Medicine, Chongqing Reproductive Genetics Institute, Chongqing Health Center for Women and Children, Women and Children's Hospital of Chongqing Medical University, Chongqing, P. R. China

8. Department of Pancreatobiliary Surgery, State Key Laboratory of Oncology in South China, Guangdong Provincial Clinical Research Center for Cancer, Sun Yat-sen University Cancer Center, Guangzhou, P. R. China

#. These authors contributed equally

***Correspondence:**

Yize Mao, maoyz@sysucc.org.cn, Department of Pancreatobiliary Surgery, State Key Laboratory of Oncology in South China, Guangdong Provincial Clinical Research Center for Cancer, Sun Yat-sen University Cancer Center, Guangzhou 510060, P. R. China

Feng Gao, gaof57@mail.sysu.edu.cn, Department of General Surgery (Colorectal Surgery), The Sixth Affiliated Hospital, Sun Yat-sen University

Xiaojian Wu, wuxjian@mail.sysu.edu.cn, Department of General Surgery (Colorectal Surgery), The Sixth Affiliated Hospital, Sun Yat-sen University

Supplementary Figure 1

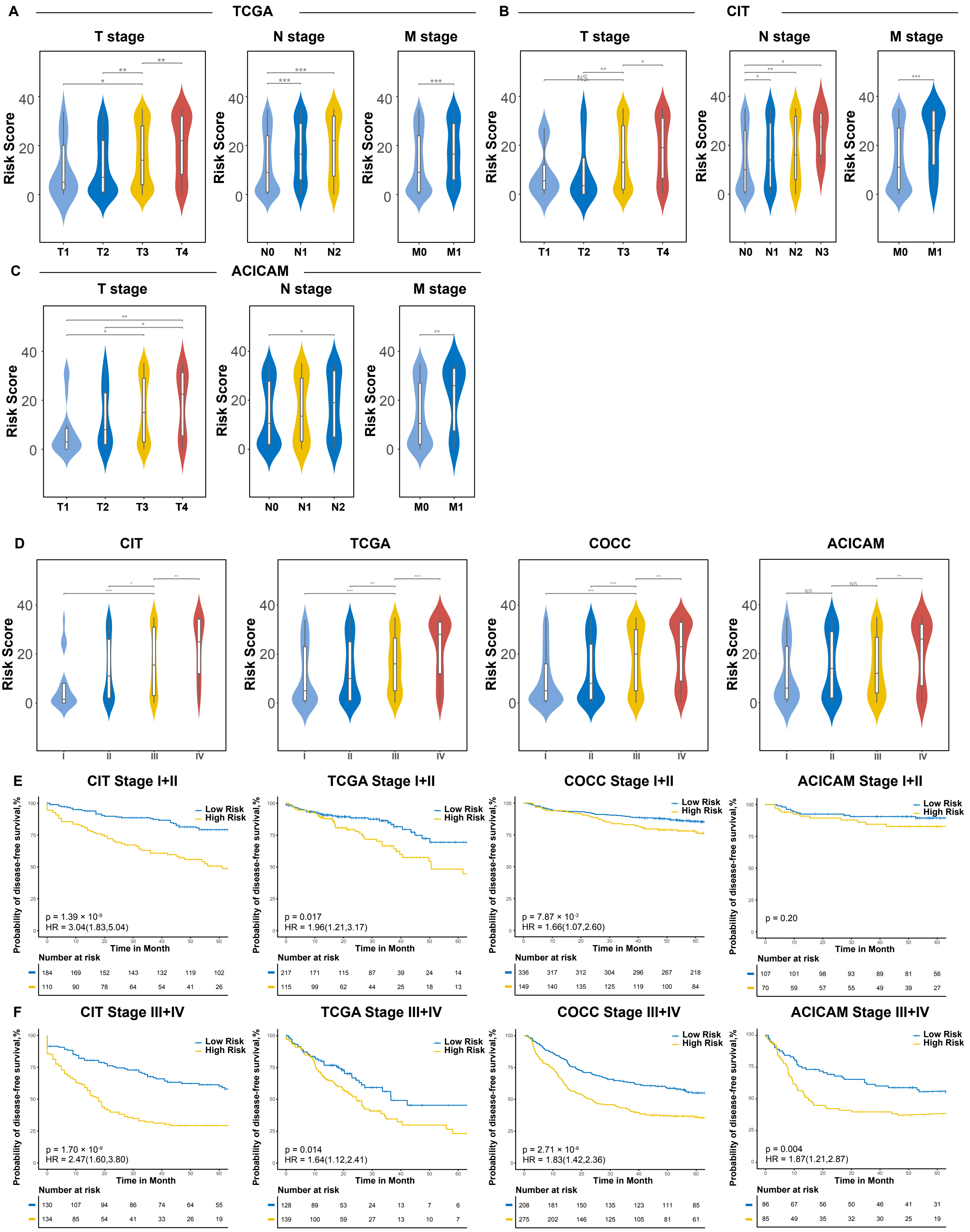

Supplementary Figure 1

(A-C) Violin plots showing associations between tumor (T), node (N), and metastasis (M) stages and risk scores in the cancer genome atlas (TCGA) (n = 618, T1 vs T3 : p = 0.04, T2 vs T3 : p = 0.0016, T3 vs T4 : p = 0.0066, N0 vs N1 : p = 8.6×10^{-6}, N0 vs N2 : p = 1.5×10^{-7}, M0 vs M1 : p = 8.6×10^{-6}), CIT(n = 562, T1 vs T3 : p = 0.06, T2 vs T3 : p = 0.0039, T3 vs T4 : p = 0.015, N0 vs N1 : p = 0.035, N0 vs N2 : p = 0.0022, N0 vs N3 : p = 0.035, M0 vs M1 : p = 1.5×10^{-6}), and ACICAM(n = 348, T1 vs T3 : p = 0.012, T1 vs T4 : p = 0.0066, T2 vs T4 : p = 0.065, N0 vs N2 : p = 0.042, M0 vs M1 : p = 0.0013).

(D) Violin plots showing the association between TNM stage and risk scores in the CIT(n = 562, I vs II : p = 0.0022, II vs III : p = 0.015, III vs IV : p = 0.0034), TCGA(n = 618, I vs III : p = 5.0×10^{-5}, II vs III : p = 0.0034, III vs IV : p = 6.1×10^{-5}), ACICAM(n = 348, I vs II : p = 0.12, II vs III : p = 0.98, III vs IV : p = 0.0049), and COCC(n = 968, I vs II : p = 0.0043, II vs III : p = 9.2×10^{-7}, III vs IV : p = 0.005).

(E-F) Kaplan-Meier curves for disease free survival (DFS) based on the PIANOS group in early- and late-stage patients in the CIT, TCGA, ACICAM, and COCC. P value was calculated with two-sided Log-Rank Test.

* p-value < 0.05, ** p-value < 0.01, *** p-value < 0.001. The box plots show the median value (centre line), the 25th and 75th percentiles (box boundaries), and the whiskers represent the data range up to 1.5 times the interquartile range from the box hinges (minima and maxima shown, excluding outliers). Outliers are not displayed. The two-sided Wilcoxon rank-sum test was utilized for intergroup comparisons. Source data are provided as a Source Data file.

Supplementary Figure 2

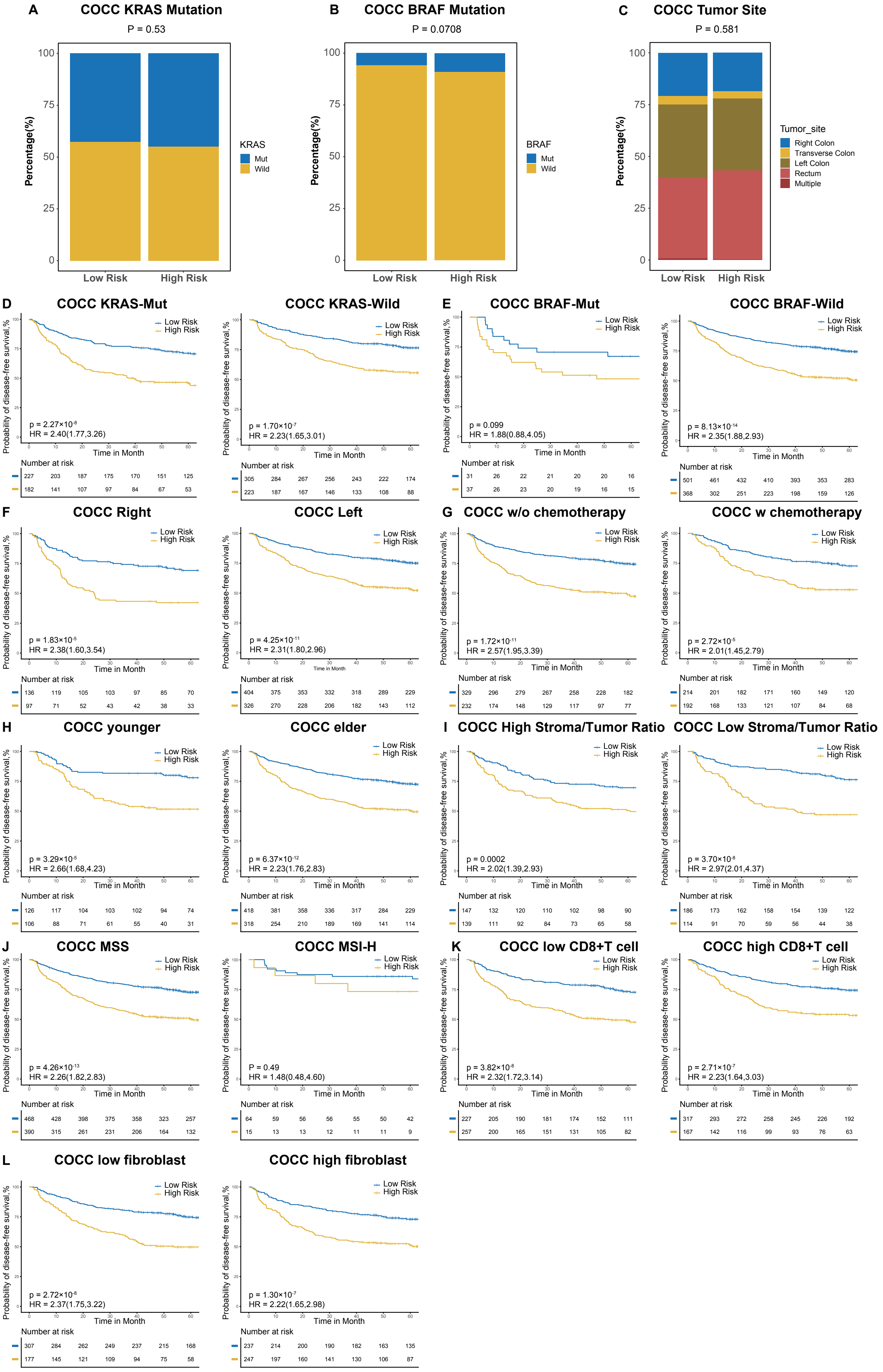

Supplementary Figure 2

(A-C) Bar plot showing differences in KRAS and BRAF mutation status(n = 937), as well as tumor location(n = 968), between high- and low-risk groups in COCC. P value was calculated with two-sided Chi-square test.

(D-L) Kaplan-Meier curves for DFS based on the PIANOS group in different KRAS and BRAF mutation statuses, tumor locations, chemotherapy status, patient age, Stroma/Tumor ratio, MSI status, CD8+ T cell and fibroblast levels in COCC. P value was calculated with two-sided Log-Rank Test.

Source data are provided as a Source Data file.

Supplementary Figure 3

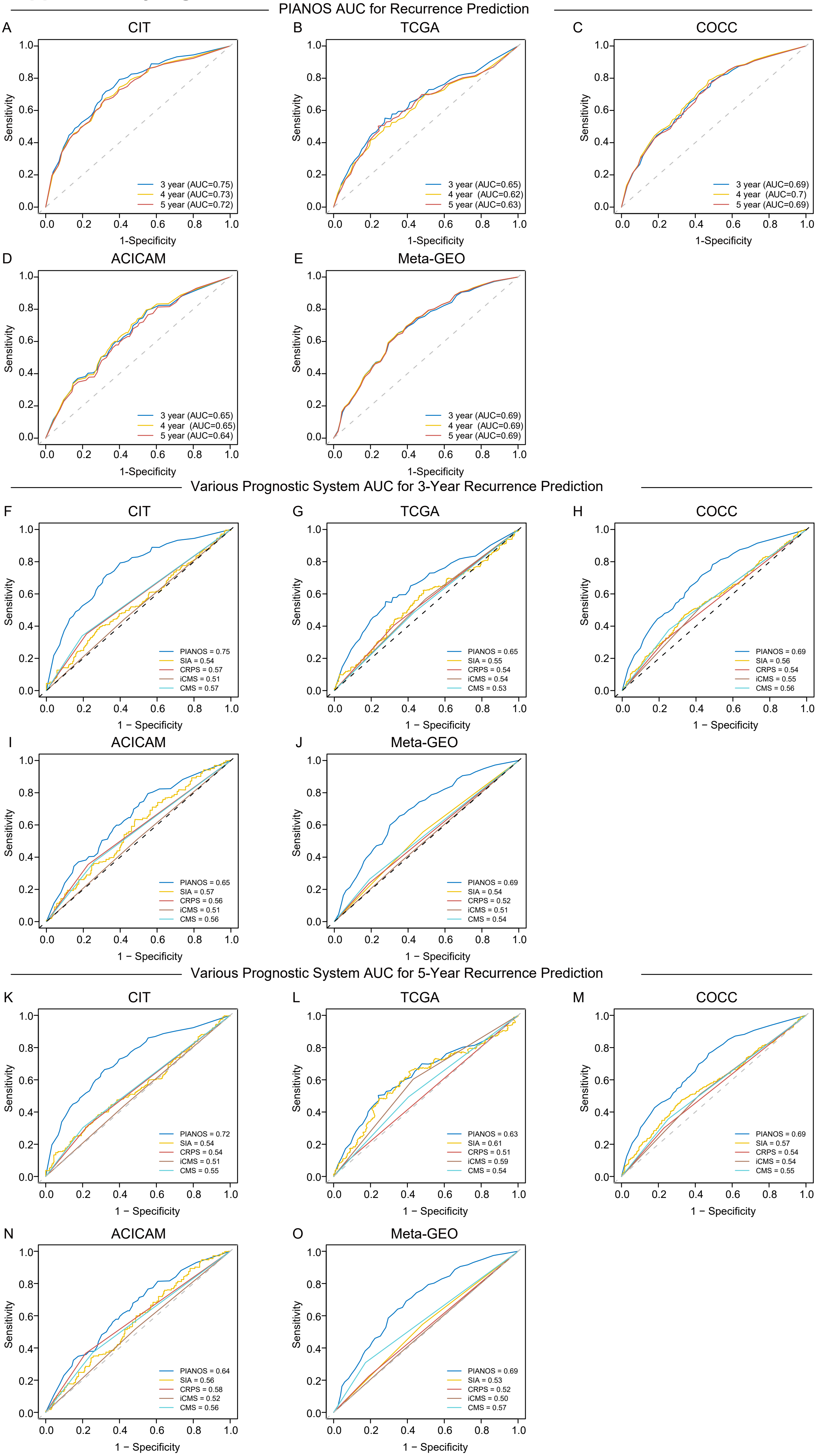

Supplementary Figure 3

(A-E) Area under the curve (AUC) for predicting 3-, 4-, and 5-year recurrence using PIANOS among patients in CIT, TCGA, COCC, ACICAM and Meta-GEO cohorts.

(F-J) AUC for predicting 3-year recurrence comparing the performance of PIANOS, iCMS, SIA, CMS and CRPS among patients in CIT, TCGA, COCC, ACICAM and Meta-GEO cohorts.

(K-O) AUC for predicting 5-year recurrence comparing the performance of PIANOS, iCMS, SIA, CMS and CRPS among patients in CIT, TCGA, COCC, ACICAM and Meta-GEO cohorts.

Datasets: CIT (n=562), COCC (n=968), TCGA (n=618), ACICAM (n=348), Meta-GEO(n = 1206). Source data are provided as a Source Data file.

Supplementary Figure 4

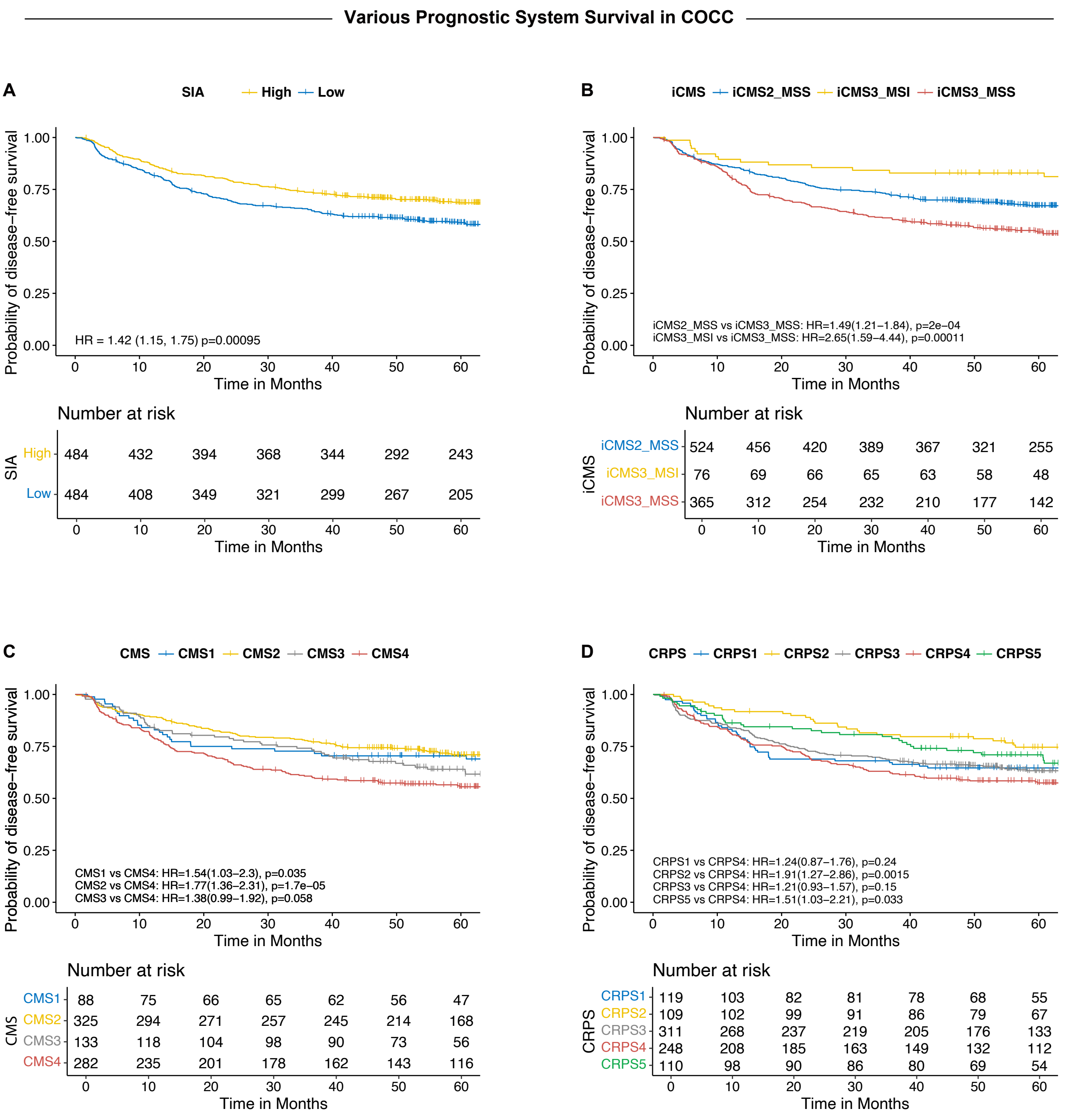

Supplementary Figure 4

(A-D) Kaplan-Meier curves for DFS based on the CMS, SIA, iCMS and CRPS in COCC(n=968). P values were calculated with two-sided log-rank test.

Supplementary Figure 5

A

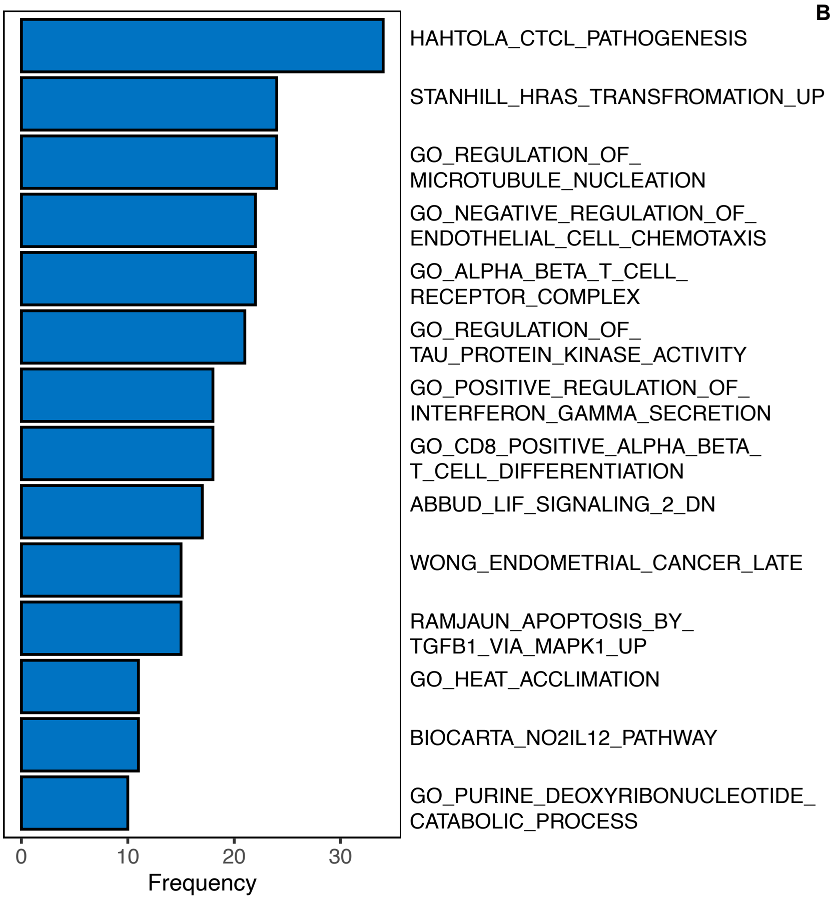

B

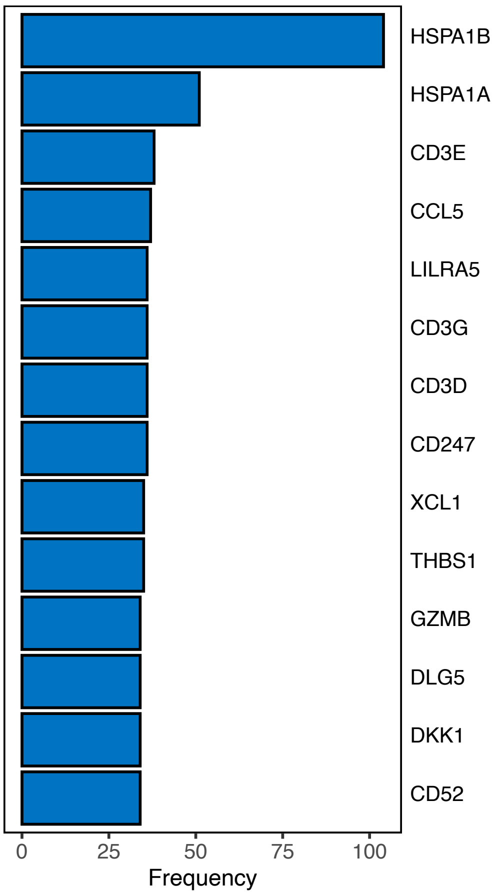

C

10% input genes

	Patient1		Patient2		Patient3	
	pathway 1	pathway 2	pathway 1	pathway 2	pathway 1	pathway 2
ssGSEA pair 1	-0.99	-0.95	0.62	1	0.99	0.98
ssGSEA pair 2	-0.77	0.69	-0.67	0.92	-0.56	0.81
ssGSEA pair 3	-0.99	-0.62	-0.69	-0.93	-0.8	-0.55
ssGSEA pair 4	-0.88	-0.53	0.57	0.53	0.83	0.85
ssGSEA pair 5	-0.7	-0.55	0.53	-0.86	-0.76	-0.7
ssGSEA pair 6	-0.53	-0.67	-0.61	-0.55	0.34	0.82
ssGSEA pair 7	-0.77	0.69	-0.51	0.92	-0.6	0.81
ssGSEA pair 8	-0.99	0.75	-0.69	-0.98	-0.8	-0.97
ssGSEA pair 9	-0.92	-1	-0.75	-0.85	-0.66	-0.99
ssGSEA pair 10	-0.77	-0.72	-0.51	0.97	-0.6	0.81
ssGSEA pair 11	-0.99	-0.95	0.62	1	0.99	0.98
ssGSEA pair 12	-0.92	-1	-0.75	-0.85	-0.66	-0.99
ssGSEA pair 13	-0.89	-0.79	-0.61	0.5	0.65	-0.7
ssGSEA pair 14	-0.96	-0.62	-0.63	-0.93	-0.92	-0.55
ssGSEA pair 15	-0.99	-0.95	0.62	1	0.99	0.98
ssGSEA pair 16	-0.77	-0.53	-0.67	0.53	-0.56	0.85
ssGSEA pair 17	-0.92	-1	-0.75	-0.85	-0.66	-0.99
ssGSEA pair 18	0.52	0.69	-0.77	0.92	-0.96	0.81
ssGSEA pair 19	-0.77	0.75	-0.51	-0.84	-0.6	-0.79
ssGSEA pair 20	-0.82	0.69	-0.72	0.92	-0.53	0.81
ssGSEA pair 21	-0.92	-1	-0.75	-0.85	-0.66	-0.99
ssGSEA pair 22	-0.99	-0.95	0.62	1	0.99	0.98
ssGSEA pair 23	-0.62	0.81	-0.87	-0.93	0.61	-0.87
ssGSEA pair 24	0.87	-0.97	-0.78	0.92	-0.7	0.97
ssGSEA pair 25	-0.99	-0.62	-0.69	-0.93	-0.8	-0.55
ssGSEA pair 26	-0.53	0.69	-0.61	0.92	0.34	0.81
ssGSEA pair 27	-0.92	-0.76	-0.75	-0.93	-0.66	-0.96
ssGSEA pair 28	-0.99	-0.95	0.62	1	0.99	0.98
ssGSEA pair 29	0.8	-0.53	-0.91	0.53	-0.92	0.85
ssGSEA pair 30	-0.94	-0.8	-0.86	-0.97	0.59	-0.53
ssGSEA pair 31	-0.63	0.75	0.57	-0.61	-0.57	0.5

50% input genes

	Patient1		Patient2		Patient3	
	pathway 1	pathway 2	pathway 1	pathway 2	pathway 1	pathway 2
ssGSEA pair 1	-0.99	-0.81	-0.61	0.56	0.72	0.95
ssGSEA pair 2	0.39	-0.56	-0.62	-0.34	-0.66	0.48
ssGSEA pair 3	-0.88	-0.61	-0.38	-0.8	-0.56	0.65
ssGSEA pair 4	-0.7	-0.45	0.38	-0.45	0.7	0.87
ssGSEA pair 5	-0.53	-0.34	-0.4	-0.7	0.64	-0.48
ssGSEA pair 6	-0.69	-0.64	-0.58	-0.52	0.57	0.29
ssGSEA pair 7	-0.65	-0.56	-0.58	-0.34	-0.53	0.48
ssGSEA pair 8	-0.88	-0.77	-0.38	-0.47	-0.56	-0.5
ssGSEA pair 9	-0.65	-0.83	-0.57	-0.52	0.64	-0.84
ssGSEA pair 10	-0.65	-0.73	-0.58	0.36	-0.53	-0.73
ssGSEA pair 11	-0.99	-0.81	-0.61	0.56	0.72	0.95
ssGSEA pair 12	-0.65	-0.83	-0.57	-0.52	0.64	-0.84
ssGSEA pair 13	-0.89	-0.79	-0.62	-0.51	0.65	-0.72
ssGSEA pair 14	-0.72	-0.61	-0.43	-0.8	-0.64	0.65
ssGSEA pair 15	-0.99	-0.81	-0.61	0.56	0.72	0.95
ssGSEA pair 16	0.39	-0.45	-0.62	-0.45	-0.66	0.87
ssGSEA pair 17	-0.65	-0.83	-0.57	-0.52	0.64	-0.84
ssGSEA pair 18	-0.93	-0.56	-0.47	-0.34	0.55	0.48
ssGSEA pair 19	-0.65	-0.73	-0.58	-0.85	-0.53	-0.42
ssGSEA pair 20	-0.47	-0.56	-0.59	-0.34	0.35	0.48
ssGSEA pair 21	-0.65	-0.83	-0.57	-0.52	0.64	-0.84
ssGSEA pair 22	-0.99	-0.81	-0.61	0.56	0.72	0.95
ssGSEA pair 23	-0.62	0.54	-0.87	-0.72	0.6	-0.55
ssGSEA pair 24	0.69	-0.65	-0.43	-0.67	0.75	0.47
ssGSEA pair 25	-0.88	-0.61	-0.38	-0.8	-0.56	0.65
ssGSEA pair 26	-0.69	-0.56	-0.58	-0.34	0.57	0.48
ssGSEA pair 27	-0.65	-0.69	-0.57	-0.84	0.64	-0.88
ssGSEA pair 28	-0.99	-0.81	-0.61	0.56	0.72	0.95
ssGSEA pair 29	-0.56	-0.45	-0.74	-0.45	0.51	0.87
ssGSEA pair 30	-0.83	-0.46	-0.78	-0.63	-0.76	-0.66
ssGSEA pair 31	-0.57	-0.63	-0.48	-0.37	-0.68	-0.67

100% input genes

	Patient1		Patient2		Patient3	
	pathway 1	pathway 2	pathway 1	pathway 2	pathway 1	pathway 2
ssGSEA pair 1	-0.95	-0.66	0.43	0.44	0.65	0.81
ssGSEA pair 2	0.36	-0.51	-0.61	-0.4	-0.66	0.46
ssGSEA pair 3	-0.81	0.53	-0.49	-0.4	-0.46	0.24
ssGSEA pair 4	-0.74	-0.32	0.28	-0.32	0.74	0.76
ssGSEA pair 5	-0.6	-0.3	-0.54	-0.66	0.49	-0.48
ssGSEA pair 6	-0.59	-0.62	-0.58	-0.55	0.44	-0.33
ssGSEA pair 7	-0.67	-0.51	-0.55	-0.4	-0.42	0.46
ssGSEA pair 8	-0.84	-0.6	-0.34	-0.57	-0.53	-0.55
ssGSEA pair 9	-0.58	-0.83	-0.58	-0.51	0.51	-0.84
ssGSEA pair 10	-0.65	-0.3	-0.52	-0.39	-0.29	-0.71
ssGSEA pair 11	-0.95	-0.66	0.43	0.44	0.65	0.81
ssGSEA pair 12	-0.58	-0.83	-0.58	-0.51	0.51	-0.84
ssGSEA pair 13	-0.8	-0.65	-0.57	-0.47	0.68	-0.51
ssGSEA pair 14	-0.59	0.53	-0.38	-0.4	-0.47	0.24
ssGSEA pair 15	-0.95	-0.66	0.43	0.44	0.65	0.81
ssGSEA pair 16	0.36	-0.32	-0.61	-0.32	-0.66	0.76
ssGSEA pair 17	-0.58	-0.83	-0.58	-0.51	0.51	-0.84
ssGSEA pair 18	-0.62	-0.51	-0.53	-0.4	0.65	0.46
ssGSEA pair 19	-0.65	-0.4	-0.52	-0.79	-0.29	-0.63
ssGSEA pair 20	-0.41	-0.51	-0.49	-0.4	0.33	0.46
ssGSEA pair 21	-0.58	-0.83	-0.58	-0.51	0.51	-0.84
ssGSEA pair 22	-0.95	-0.66	0.43	0.44	0.65	0.81
ssGSEA pair 23	-0.71	0.4	-0.66	-0.63	0.56	-0.45
ssGSEA pair 24	-0.49	-0.65	-0.52	-0.69	0.56	0.33
ssGSEA pair 25	-0.81	0.53	-0.49	-0.4	-0.46	0.24
ssGSEA pair 26	-0.59	-0.51	-0.58	-0.4	0.44	0.46
ssGSEA pair 27	-0.58	-0.36	-0.58	-0.81	0.51	-0.7
ssGSEA pair 28	-0.95	-0.66	0.43	0.44	0.65	0.81
ssGSEA pair 29	-0.56	-0.32	-0.62	-0.32	0.61	0.76
ssGSEA pair 30	-0.82	-0.74	-0.78	-0.58	-0.78	-0.46
ssGSEA pair 31	-0.6	-0.49	-0.52	-0.44	-0.71	-0.62

Higher ssGSEA in TSP

Lower ssGSEA in TSP

Supplementary Figure 5

(A) Highest frequency pathways identified in PIANOS.

(B) Highest frequency genes identified in PIANOS.

(C) Heatmap of ssGSEA scores showing different proportions of gene deletions

Supplementary Figure 6

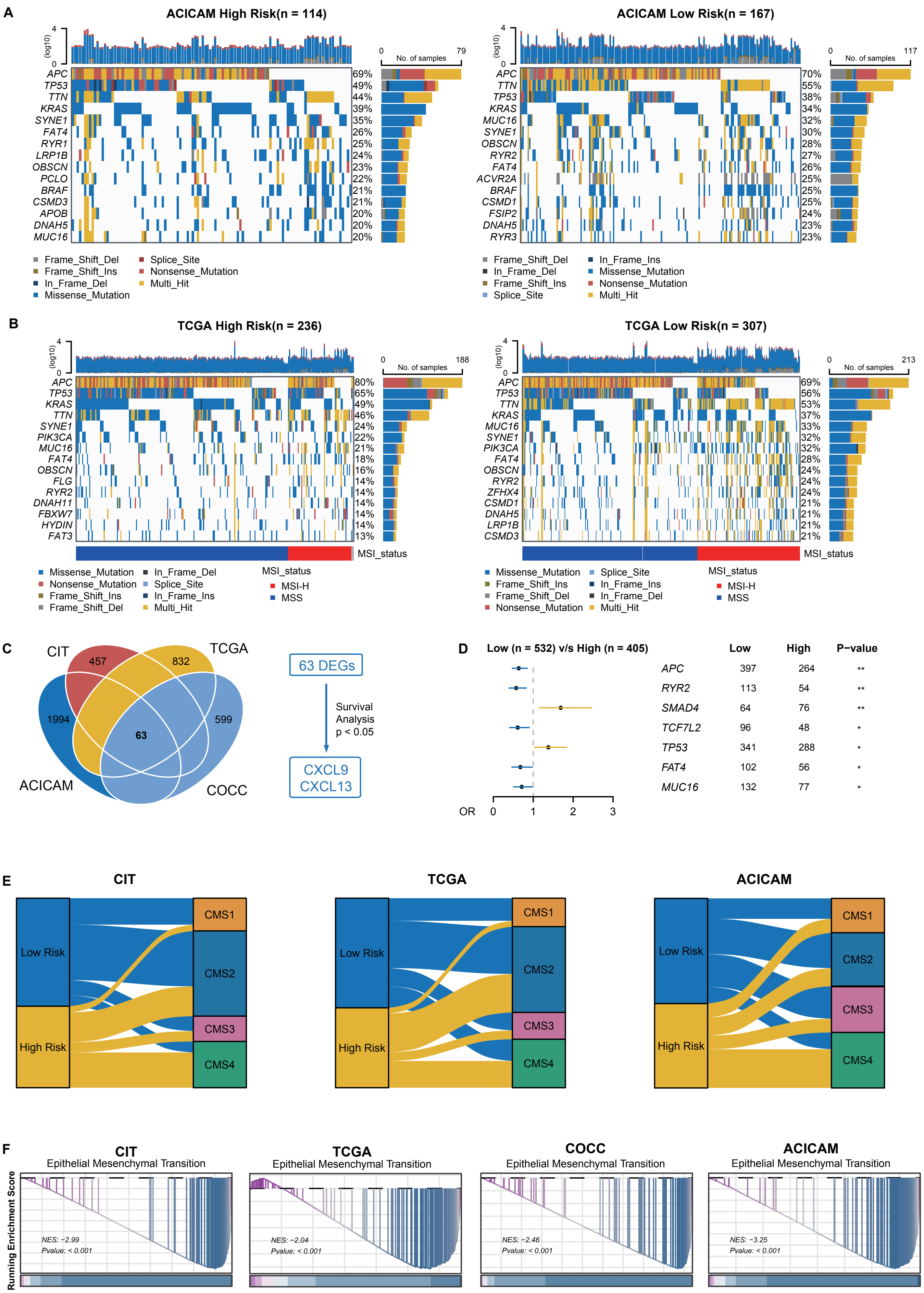

Supplementary Figure 6

(A, B) Waterfall plot showing the top 15 gene mutations with the highest frequency among the PIANOS groups in the ACICAM and TCGA cohorts.

(C) Venn diagram illustrating the screening process for CXCL9 and CXCL13 genes.

(D) High-frequency mutated genes with differential frequencies between COCC high- and low-risk groups(APC $p = 0.0022$, RYR2 $p = 0.0023$, SMAD4 $p = 0.0056$, TCF7L2 $p = 0.012$, TP53 $p = 0.028$, FAT4 $p = 0.038$, MUC16 $p = 0.042$). P values of bar plot was calculated with two-sided Chi-square test.

(E) Sankey diagram comparing the PIANOS groups and CMS subtypes.

(F) Gene set enrichment analysis (GSEA) plots of the epithelial-mesenchymal transition pathway for low-versus high-risk in CIT($p < 1\times10^{-10}$), TCGA($p < 1\times10^{-10}$), ACICAM($p < 1\times10^{-10}$), and COCC($p < 1\times10^{-10}$). P value was calculated with two-sided Permutation Test.

Datasets: CIT (n=562), COCC (n=968), TCGA (n=618), ACICAM (n=348).

Supplementary Figure 7

A

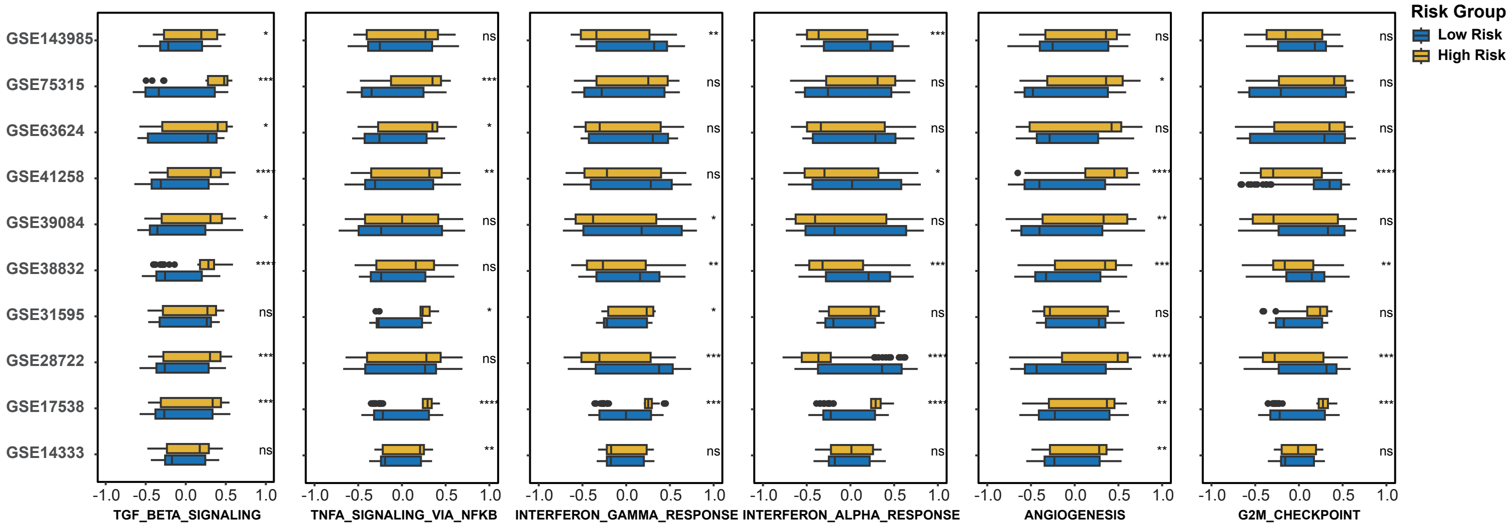

B

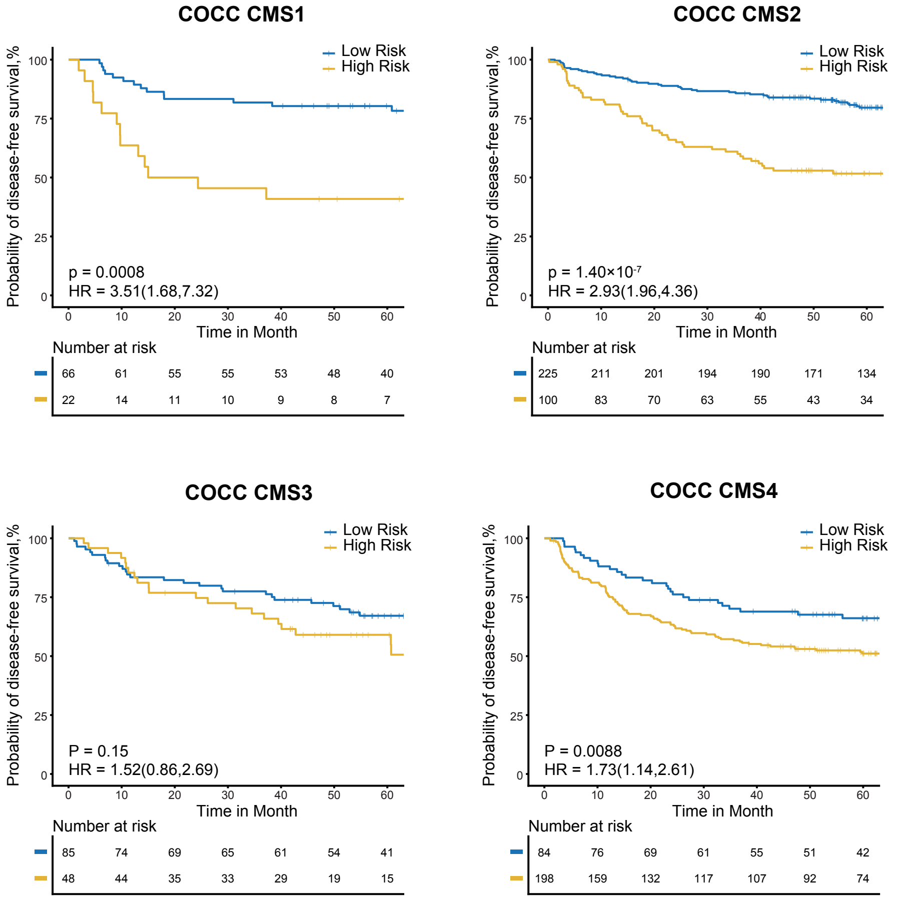

Supplementary Figure 7

(A) Bar plot comparing the enrichment scores of six selected pathways between the PIANOS groups in the 10 GEO cohorts. * p-value < 0.05, ** p-value < 0.01, *** p-value < 0.001.The box plots show the median value (centre line), the 25th and 75th percentiles (box boundaries), and the whiskers represent the data range up to 1.5 times the interquartile range from the box hinges (minima and maxima shown). Outliers are displayed. Exact p values are shown in Supplementary Data 3D. Datasets: GSE14333 n = 226, GSE17538 n = 200, GSE41258 n = 142, GSE28722 n = 125, GSE38832 n = 122, GSE143985 n = 91, GSE39084 n = 67, GSE63624 n = 49, GSE75315 n = 49, GSE31595 n = 37.

(B) Kaplan-Meier curves for DFS based on the PIANOS group in different CMS subtypes in COCC. P values were calculated using the two-sided log-rank test.

Source data are provided as a Source Data file.

Supplementary Figure 8

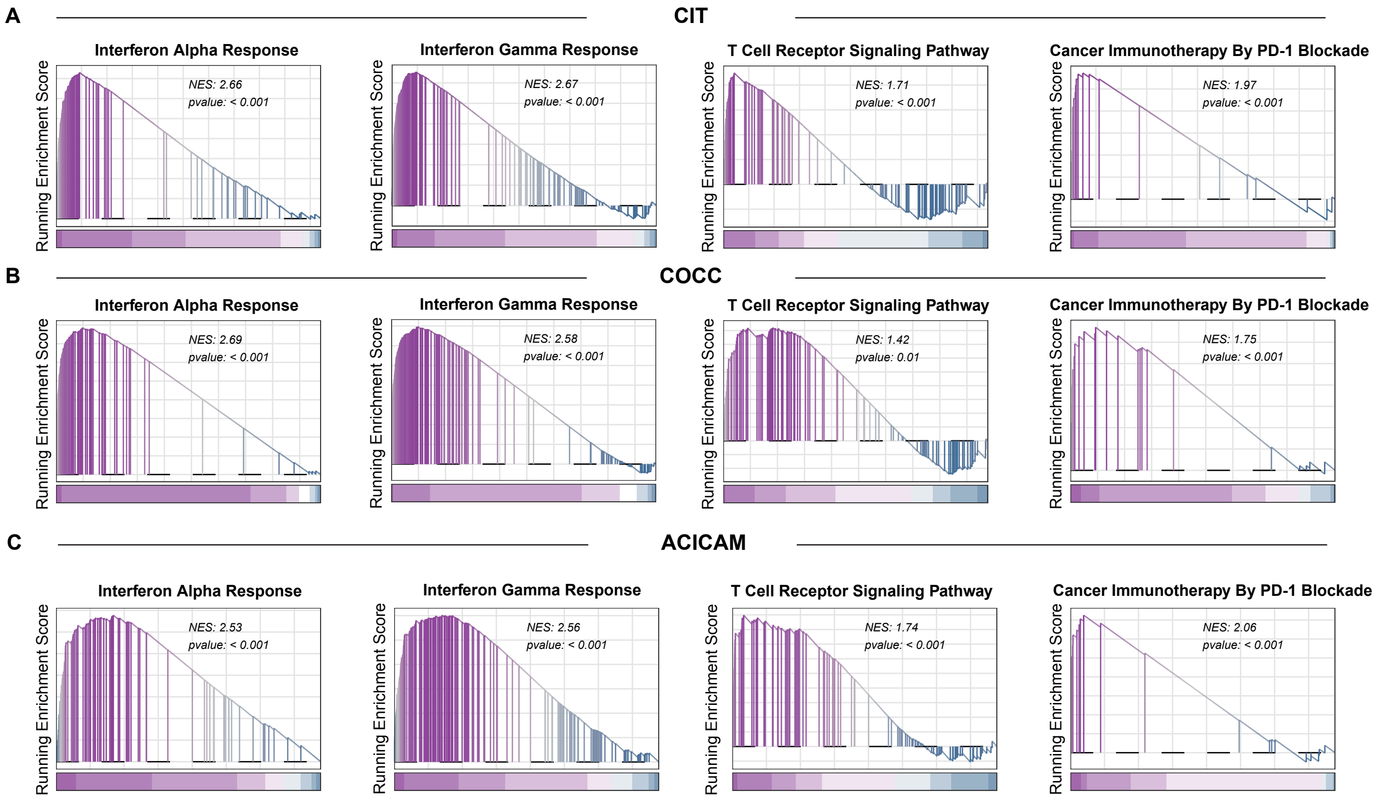

Supplementary Figure 8

(A, B, C) GSEA plots of interferon (IFN)-α response(CIT $p < 1\times10^{-10}$, COCC $p < 1\times10^{-10}$, ACICAM $p < 1\times10^{-10}$) and interferon (IFN)-γ response(CIT $p < 1\times10^{-10}$, COCC $p < 1\times10^{-10}$, ACICAM $p < 1\times10^{-10}$), T cell receptor signaling pathways(CIT $p = 7.00\times10^{-5}$, COCC $p = 0.01$, ACICAM $p = 1.43\times10^{-4}$), and cancer immunotherapy by programmed cell-death 1 (PD-1) pathway(CIT $p = 4.28\times10^{-3}$, COCC $p = 2.15\times10^{-4}$, ACICAM $p = 6.18\times10^{-5}$) between low-risk versus high-risk in the CIT, ACICAM and COCC. P value was calculated with two-sided Permutation Test.

Datasets: CIT (n=562), COCC (n=968), ACICAM (n=348)

Supplementary Note 1

Data Access Agreement

The Sixth Affiliated Hospital (SAH) of Sun Yat-sen University and the Recipient Institution (RECIPIENT) hereby enter into this Agreement for the transfer of data used in the paper by ____ ___________Author and Title ________________

In consideration of SAH providing data to RECIPIENT, RECIPIENT hereby agrees to the following terms and conditions:

1. Data will be provided to RECIPIENT with a Research Plan that is approved by SAH.
2. Data will be used only by RECIPIENT for purpose described in the Research Plan.
3. The RECIPIENT will not release data to a third party without prior approval from SAH.
4. The RECIPIENT will not share, publish, or otherwise release any findings or conclusions derived from analysis of data obtained from SAH without prior approval from SAH.
5. All data transferred to RECIPIENT shall remain the property of SAH.

____

For the Sixth Affiliated Hospital of Sun Yat-sen University

Date: _______________________________

____

For the RECIPIENT

Date: _______________________________
