# Supplementary material for: Personalized risk stratification in colorectal cancer via PIANOS system
Source: Nat Commun. 2025 Jul 16;16:6561. doi: 10.1038/s41467-025-61713-1 (PMC12267411; doi:10.1038/s41467-025-61713-1)
Supplement: Supplementary file 3 — Description of Additional Supplementary Files [file 41467_2025_61713_MOESM3_ESM.pdf]

Description of Additional Supplementary Files

Supplementary Data 1

Baseline information of CIT. P-values correspond to comparisons across Risk Groups using appropriate statistical tests (Kruskal-Wallis for continuous, Chi-squared for categorical variables). Tests were two-sided, and no correction for multiple variable testing was applied.

Supplementary Data 2

Baseline information of TCGA. P-values correspond to comparisons across Risk Groups using appropriate statistical tests (Kruskal-Wallis for continuous, Chi-squared for categorical variables). Tests were two-sided, and no correction for multiple variable testing was applied.

Supplementary Data 3

Baseline information of COCC. P-values correspond to comparisons across Risk Groups using appropriate statistical tests (Kruskal-Wallis for continuous, Chi-squared for categorical variables). Tests were two-sided, and no correction for multiple variable testing was applied.

Supplementary Data 4

Univariate Cox regression analysis of risk factors for DFS in CIT. P-values are derived from two-sided Wald tests. Multiple comparison adjustments were not performed.

Supplementary Data 5

Univariate Cox regression analysis of risk factors for DFS in TCGA. P-values are derived from two-sided Wald tests. Multiple comparison adjustments were not performed.

Supplementary Data 6

Univariate Cox regression analysis of risk factors for DFS in COCC. P-values are derived from two-sided Wald tests. Multiple comparison adjustments were not performed.

Supplementary Data 7

Multivariate Cox regression analysis of risk factors for DFS in CIT. P-values are derived from two-sided Wald tests. Multiple comparison adjustments were not performed.

Supplementary Data 8

Multivariate Cox regression analysis of risk factors for DFS in TCGA. P-values are derived from two-sided Wald tests. Multiple comparison adjustments were not performed.

Supplementary Data 9

Multivariate Cox regression analysis of risk factors for DFS in COCC. P-values are derived from two-sided Wald tests. Multiple comparison adjustments were not performed.

Supplementary Data 10

Proportions of high- and low-risk PIANOS groups in all cohorts.

Supplementary Data 11

C-Index of PIANOS in CRC validation cohorts. Analysis conducted across all cohorts, and the meta-estimates for sequencing cohorts, array-based cohorts, and both platforms combined. Significance relative to reference values (C-index=0.5) can be inferred from the 95% CIs. No multiple comparison adjustments were applied.

Supplementary Data 12

D-Index of PIANOS in CRC validation cohorts Analysis conducted across all cohorts, and the meta-estimates for sequencing cohorts, array-based cohorts, and both platforms combined. Significance for the D-index is based on the two-sided log-rank test, while significance relative to reference values (D-index=1) can be inferred from the 95% CIs. No multiple comparison adjustments were applied.

Supplementary Data 13

Expression pattern template of iCMS marker genes.

Supplementary Data 14

Details of 105 CRC models.

Supplementary Data 15

Proportion of different CMS subtypes in high- and low-risk PIANOS groups in CIT.

Supplementary Data 16

Proportion of different CMS subtypes in high- and low-risk PIANOS groups in TCGA.

Supplementary Data 17

Proportion of different CMS subtypes in high- and low-risk PIANOS groups in COCC.

Supplementary Data 18

Exact p-Values Comparing Enrichment Scores of Six Key Pathways between PIANOS Risk Groups in 10 GEO Cohorts

Supplementary Data 19

Baseline information of the patients with stage III and IV in low- Ki-67 groups in COCC.

Supplementary Data 20

Baseline information of the patients with stage III and IV in high- Ki-67 groups in COCC.

Supplementary Data 21

Baseline information of the patients with stage III and IV in high-risk groups in COCC.

Supplementary Data 22

Baseline information of the patients with stage III and IV in low-risk groups in COCC.

Supplementary Data 23
Baseline information for patients in GSE104645 cohorts.
P-values correspond to comparisons across Risk Groups using appropriate statistical tests (Kruskal-Wallis for continuous, Chi-squared for categorical variables). Tests were two-sided, and no correction for multiple variable testing was applied.

Supplementary Data 24
Baseline information for patients in GSE87211 cohorts.
P-values correspond to comparisons across Risk Groups using appropriate statistical tests (Kruskal-Wallis for continuous, Chi-squared for categorical variables). Tests were two-sided, and no correction for multiple variable testing was applied.

Supplementary Data 25
Baseline information of stage IV patients with and without Bevacizumab in COCC with low expression of VEGFA.

Supplementary Data 26
Baseline information of stage IV patients with and without Bevacizumab in COCC with high expression of VEGFA.

Supplementary Data 27
Baseline information of stage IV patients with and without Bevacizumab in low-risk in COCC.
P-values correspond to comparisons across Risk Groups using appropriate statistical tests (Kruskal-Wallis for continuous, Chi-squared for categorical variables). Tests were two-sided, and no correction for multiple variable testing was applied.

Supplementary Data 28
Baseline information of stage IV patients with and without Bevacizumab in high-risk in COCC.
P-values correspond to comparisons across Risk Groups using appropriate statistical tests (Kruskal-Wallis for continuous, Chi-squared for categorical variables). Tests were two-sided, and no correction for multiple variable testing was applied.

Supplementary Data 29
Detailed information on immune-cell counts predicted by deep learning on pathology images in high- and low-risk groups.

Supplementary Data 30
Detailed information on immune cell abundance, immunomodulatory gene expression, and immune checkpoint gene expression in low-risk group compared with those of low-risk group. Significance was assessed by the two-sided Wilcoxon rank-sum test. P-values are uncorrected for multiple comparisons.

Supplementary Data 31
List of cohorts, software and algorithms used in this study.
