# Supplementary material for: Personalized risk stratification in colorectal cancer via PIANOS system
Source: Nat Commun. 2025 Jul 16;16:6561. doi: 10.1038/s41467-025-61713-1 (PMC12267411; doi:10.1038/s41467-025-61713-1)
Supplement: Supplementary file 5 — Reporting Summary [file 41467_2025_61713_MOESM5_ESM.pdf]

nature portfolio

Corresponding author(s): Yize Mao, Feng Gao, Xiaojian Wu

Last updated by author(s): May 16, 2025

Reporting Summary

Nature Portfolio wishes to improve the reproducibility of the work that we publish. This form provides structure for consistency and transparency in reporting. For further information on Nature Portfolio policies, see our Editorial Policies and the Editorial Policy Checklist.

Statistics

For all statistical analyses, confirm that the following items are present in the figure legend, table legend, main text, or Methods section.

n/a	Confirmed	
☐	☒	The exact sample size (*n*) for each experimental group/condition, given as a discrete number and unit of measurement
☒	☐	A statement on whether measurements were taken from distinct samples or whether the same sample was measured repeatedly
☐	☒	The statistical test(s) used AND whether they are one- or two-sided<br>*Only common tests should be described solely by name; describe more complex techniques in the Methods section.*
☐	☒	A description of all covariates tested
☐	☒	A description of any assumptions or corrections, such as tests of normality and adjustment for multiple comparisons
☐	☒	A full description of the statistical parameters including central tendency (e.g. means) or other basic estimates (e.g. regression coefficient) AND variation (e.g. standard deviation) or associated estimates of uncertainty (e.g. confidence intervals)
☐	☒	For null hypothesis testing, the test statistic (e.g. *F*, *t*, *r*) with confidence intervals, effect sizes, degrees of freedom and *P* value noted<br>*Give P values as exact values whenever suitable.*
☒	☐	For Bayesian analysis, information on the choice of priors and Markov chain Monte Carlo settings
☒	☐	For hierarchical and complex designs, identification of the appropriate level for tests and full reporting of outcomes
☐	☒	Estimates of effect sizes (e.g. Cohen's *d*, Pearson's *r*), indicating how they were calculated

Our web collection on statistics for biologists contains articles on many of the points above.

Software and code

Policy information about availability of computer code

Data collection	GEO data were downloaded using the R package GEOquery (Version: 2.68.0) in R (Version 4.3.1).
Data analysis	The data analyses were performed using R (Version 4.3.1) and the following R packages: affy (Version: 1.78.2), caret (Version: 6.0-94), clusterProfiler (Version: 4.8.3), compareGroups (Version: 4.7.2), ComplexHeatmap (Version: 2.16.0), Cowplot (Version: 1.1.2), DeepCC (Version: 1.48.3), dplyr (Version: 1.1.4), foreign (Version: 0.8-86), forestplot (Version: 3.1.3), GEOquery (Version: 2.68.0), ggalluvial (Version: 0.12.5), ggExtra (Version: 0.10.1), ggplot2 (Version: 3.4.4), ggpubr (Version: 0.6.0), ggrepel (Version: 0.9.4), ggsci (Version: 3.0.0), ggstatsplot (Version: 0.12.1), GseaVis (Version: 0.0.9), GSVA (Version: 1.48.3), gtools (Version: 3.9.5), IOBR (Version: 0.99.9), jstable (Version: 1.1.3), limma (Version: 3.56.2), maftools (Version: 2.16.0), magrittr (Version: 2.0.3), maps (Version: 3.4.2), MASS (Version: 7.3-60), MatchIt (Version: 4.5.5), modEvA (Version: 3.11), msigdbr (Version: 7.5.1), oncoPredict (Version: 0.2), org.Hs.eg.db (Version: 3.17.0), plyr (Version: 1.8.9), pROC (Version: 1.18.5), randomForestSRC (Version: 3.2.3), RColorBrewer (Version: 1.1-3), readxl (Version: 1.4.3), reshape2 (Version: 1.4.4), RISCA (Version: 1.0.4), Rmisc (Version: 1.5.1), rstatix (Version: 0.7.2), stringr (Version: 1.5.1), survcomp (Version: 1.50.0), survival (Version: 3.5-7), survminer (Version: 0.4.9), switchBox (Version: 1.36.0), tableone (Version: 0.13.2), tidyverse (Version: 2.0.0), timeROC (Version: 0.4), tinyarray (Version: 2.3.1), visdat (Version: 0.6.0), waterfalls (Version: 1.0.0), and xCell (Version: 1.1.0).

For manuscripts utilizing custom algorithms or software that are central to the research but not yet described in published literature, software must be made available to editors and reviewers. We strongly encourage code deposition in a community repository (e.g. GitHub). See the Nature Portfolio guidelines for submitting code & software for further information.

Data

Policy information about availability of data

All manuscripts must include a data availability statement. This statement should provide the following information, where applicable:

- Accession codes, unique identifiers, or web links for publicly available datasets
- A description of any restrictions on data availability
- For clinical datasets or third party data, please ensure that the statement adheres to our policy

Gene data and clinical information from all public datasets are available online, with the URLs mentioned in the Supplementary Table 7, including ACICAM [https://www.ncbi.nlm.nih.gov/pmc/articles/PMC10202816/]: Datasets of RNA-Seq, Whole-Exome Sequencing, TCR sequencing of 348 CRC samples and adjacent normal tissue samples, TCGA [https://portal.gdc.cancer.gov/]: Datasets of RNA-Seq, Whole-genome Sequencing of 633 CRC samples and adjacent normal tissue samples, IMvigor210 [https://pubmed.ncbi.nlm.nih.gov/29443960/]: RNA-seq of 298 pre-treatment tumour tissue samples from metastatic urothelial carcinoma patients treated with atezolizumab, NIHMS1737783 [https://www.ncbi.nlm.nih.gov/pmc/articles/PMC8493486/]: Whole-exome sequencing and mRNA-seq of 886 baseline tumour tissue samples from advanced renal cell carcinoma patients, PRJEB23709 [https://linkinghub.elsevier.com/retrieve/pii/S1535-6108(19)30037-6]: RNA-seq of 158 tumor biopsy specimens from metastatic melanoma patients, PRJEB25780 [https://www.nature.com/articles/s41591-018-0101-z]: RNA-seq of 61 pretreatment tumor tissue samples from metastatic gastric cancer patients treated with pembrolizumab, GSE104645 [https://www.ncbi.nlm.nih.gov/geo/query/acc.cgi?acc=GSE104645]: Expression profiling by array of 193 formalin-fixed, paraffin-embedded primary colorectal cancer tumor samples, GSE31595 [https://www.ncbi.nlm.nih.gov/geo/query/acc.cgi?acc=GSE31595]: Expression profiling by array of 37 stage II and III colon cancer tumor samples, GSE39582 [https://www.ncbi.nlm.nih.gov/geo/query/acc.cgi?acc=GSE39582]: Expression profiling by array of 585 colorectal tissue samples, GSE35640 [https://www.ncbi.nlm.nih.gov/geo/query/acc.cgi?acc=GSE35640]: Expression profiling by array of 65 pre-treatment tumor biopsy samples from metastatic melanoma and early-stage non-small-cell lung cancer patients, GSE14333 [https://www.ncbi.nlm.nih.gov/geo/query/acc.cgi?acc=GSE14333]: Expression profiling by array of 290 primary colorectal cancer tumor samples, GSE39084 [https://www.ncbi.nlm.nih.gov/geo/query/acc.cgi?acc=GSE39084]: Expression profiling by array of 70 primary colorectal cancer tumor tissue samples, GSE28722 [https://www.ncbi.nlm.nih.gov/geo/query/acc.cgi?acc=GSE28722]: Expression profiling by two-color microarray of 129 primary colorectal tumor tissue samples hybridized against a pooled common reference, GSE41258 [https://www.ncbi.nlm.nih.gov/geo/query/acc.cgi?acc=GSE41258]: Expression profiling by array of 390 colorectal patient tissue samples, GSE178341 [https://www.ncbi.nlm.nih.gov/geo/query/acc.cgi?acc=GSE178341]: Single-cell RNA-seq of 371,223 dissociated cells from primary, treatment-naïve colorectal cancer tumors and adjacent normal mucosa, spanning 28 mismatch-repair proficient and 34 mismatch-repair deficient patients, GSE17538 [https://www.ncbi.nlm.nih.gov/geo/query/acc.cgi?acc=GSE17538]: Expression profiling by array of 232 primary colorectal cancer tumor samples, GSE38832 [https://www.ncbi.nlm.nih.gov/geo/query/acc.cgi?acc=GSE38832]: Expression profiling by array of 122 primary colorectal cancer tumor tissue samples, GSE63624 [https://www.ncbi.nlm.nih.gov/geo/query/acc.cgi?acc=GSE63624]: Expression profiling by exon microarray of 52 primary proximal colon cancer tumor tissue samples, GSE75315 [https://www.ncbi.nlm.nih.gov/geo/query/acc.cgi?acc=GSE75315]: Expression profiling by exon microarray of 211 primary colorectal cancer tumor tissue samples, GSE136961 [https://www.ncbi.nlm.nih.gov/geo/query/acc.cgi?acc=GSE136961]: Targeted RNA sequencing of 21 pre-treatment non–small cell lung cancer tumor tissue samples, GSE91061 [https://www.ncbi.nlm.nih.gov/geo/query/acc.cgi?acc=GSE91061]: RNA-seq of 118 tumor biopsy samples from 65 melanoma patients, GSE143985 [https://www.ncbi.nlm.nih.gov/geo/query/acc.cgi?acc=GSE143985]: Expression profiling by array of 91 primary colorectal tumour samples and GSE87211 [https://www.ncbi.nlm.nih.gov/geo/query/acc.cgi?acc=GSE87211]: Expression profiling by array of 363 rectal cancer and matched mucosa samples from 243 patients. The sequencing data and associated clinical information for the COCC cohort generated in this study have been deposited in the China National Center for Bioinformation (CNCB) BioProject database under accession code HRA007315 [https://ngdc.cncb.ac.cn/gsa-human/browse/HRA007315]: Datasets of genome, transcriptome, epigenetics of 1050 CRC samples and adjacent normal tissue samples, DNBSEQ-T1×5RS. Due to patient privacy regulations and legal restrictions regarding the sharing of human genomic data, access to the COCC cohort data is available under restricted access. Access can be obtained by contacting the corresponding author, Dr. Xiaojian Wu(wuxjian@mail.sysu.edu.cn), with an appropriate data sharing agreement. Source data are provided as a Source Data file.

Research involving human participants, their data, or biological material

Policy information about studies with human participants or human data. See also policy information about sex, gender (identity/presentation), and sexual orientation and race, ethnicity and racism.

Reporting on sex and gender	For the COCC cohort, participant sex was determined retrospectively from hospital medical records, based on the legal sex registered in each individual's national Resident Identity Card in China. Gender identity beyond this binary classification was not assessed. Because our primary objective was to build a prognostic model broadly applicable to all colorectal cancer patients, we did not perform sex-specific subgroup analyses.
Reporting on race, ethnicity, or other socially relevant groupings	We do not analyze the patient's race, ethnicity or socially relevant groupings.
Population characteristics	We obtained gene expression profiles and follow-up data of 3666 patients with colorectal cancer from 15 cohorts to validate PIANOS, including the Gene Expression Omnibus (GEO), The Cancer Genome Atlas (TCGA), and in-house Clinical Omics Study of Colorectal Cancer in China (COCC) CRC cohorts. Moreover, we collected 396 patients with CRC from neoadjuvant therapy cohorts to predict neoadjuvant therapy efficacy. We also collected seven immunotherapy cohorts with 1377 patients to explore the predictive value of PIANOS on immunotherapy response. Detailed population characteristics of cohorts used in this study can be found in Supplementary Table1.
Recruitment	For the COCC CRC cohort, all patients were pathologically diagnosed as colorectal cancer in the Sixth Affiliated Hospital of Sun Yat-sen University and the samples were collected with informed consent. Written informed consent was obtained from all participants prior to sample collection.<br>For other cohorts, the outliers and exclusion criteria can be accessed using the above link (Data part).
Ethics oversight	For Clinical Omics Study of Colorectal Cancer in China (COCC) CRC cohort, the Institutional Ethical Review Boards of the Sixth Affiliated Hospital, Sun Yat-sen University approved this study (2024ZSLYEC-408).<br>For other public cohorts, the ethics was not applicable.

Note that full information on the approval of the study protocol must also be provided in the manuscript.

Field-specific reporting

Please select the one below that is the best fit for your research. If you are not sure, read the appropriate sections before making your selection.

☒ Life sciences ☐ Behavioural & social sciences ☐ Ecological, evolutionary & environmental sciences

For a reference copy of the document with all sections, see nature.com/documents/nr-reporting-summary-flat.pdf

Life sciences study design

All studies must disclose on these points even when the disclosure is negative.

Sample size	Sample sizes for the cohorts used in this study were primarily determined by the availability of public datasets or the number of patients enrolled in our in-house COCC cohort during the study period. No formal statistical method was used to predetermine sample size prior to data collection. However, the substantial total sample size across all development and validation cohorts (totaling n=3666) provided robust statistical power for model development, validation, and assessment of its generalizability.
Data exclusions	Patients with mismatched gene expression data and clinical information were excluded from the analyses to ensure data integrity.
Replication	During the development of the k-TSP classifier, the model building process was repeated 100 times, and models achieving a balanced accuracy greater than 0.6 were selected for inclusion in the final PIANOS model to ensure robustness and optimal performance. The final PIANOS model, developed on the CIT cohort, was subsequently rigorously validated for its prognostic performance and generalizability on multiple independent external cohorts, including the COCC, TCGA, ACICAM, and meta-GEO cohorts.<br>To assess the independent prognostic value of the PIANOS stratification, multivariate Cox proportional hazards regression analyses were performed. Covariates included in these models were selected using a forward stepwise Akaike Information Criterion (stepAIC) approach, considering known clinical and pathological prognostic factors.
Randomization	No randomization was performed for primary analysis of the clinical trial data in the usual meaning of 'randomization in clinical trials'.
Blinding	No blinding was performed, The clinical data analysis is retrospective and thus no blinding was performed.

Reporting for specific materials, systems and methods

We require information from authors about some types of materials, experimental systems and methods used in many studies. Here, indicate whether each material, system or method listed is relevant to your study. If you are not sure if a list item applies to your research, read the appropriate section before selecting a response.

Materials & experimental systems

n/a	Involved in the study	
☐	☒	Antibodies
☒	☐	Eukaryotic cell lines
☒	☐	Palaeontology and archaeology
☒	☐	Animals and other organisms
☒	☐	Clinical data
☒	☐	Dual use research of concern
☒	☐	Plants

Methods

n/a	Involved in the study	
☒	☐	ChIP-seq
☒	☐	Flow cytometry
☒	☐	MRI-based neuroimaging

Antibodies

Antibodies used	Paraffin-embedded tumour tissue sections were dried at 65°C for 15 min and then placed in an automatic IHC machine (BenchMark XT, Roche) for staining. The following primary antibodies were used: MLH1 (mouse monoclonal, clone ES05, Cat No. MAB-0789, MXB), MSH2 (rabbit monoclonal, clone LBP2-MSH2, Cat No. IR376, LBP), MSH6 (rabbit monoclonal, clone EP49, Cat No. ZA-0541, ZSGB-BIO), PMS2 (rabbit monoclonal, clone EP51, Cat No. ZA-0542, ZSGB-BIO), and Ki67 (mouse monoclonal, clone MIB-1, Cat No. IR62661-2, Dako). All antibodies were ready-to-use formulations and applied according to the manufacturer's instructions within the automated system.
Validation	All primary antibodies used are commercially available and validated by the manufacturers for immunohistochemistry (IHC) on FFPE tissues. They are routinely used for clinical diagnostic purposes in accredited pathology laboratories, which further supports their validation for the targets specified. This information is implicitly covered by their use as ready-to-use diagnostic reagents according to manufacturer guidelines.

Plants

Seed stocks	*Report on the source of all seed stocks or other plant material used. If applicable, state the seed stock centre and catalogue number. If plant specimens were collected from the field, describe the collection location, date and sampling procedures.*
Novel plant genotypes	*Describe the methods by which all novel plant genotypes were produced. This includes those generated by transgenic approaches, gene editing, chemical/radiation-based mutagenesis and hybridization. For transgenic lines, describe the transformation method, the number of independent lines analyzed and the generation upon which experiments were performed. For gene-edited lines, describe the editor used, the endogenous sequence targeted for editing, the targeting guide RNA sequence (if applicable) and how the editor was applied.*
Authentication	*Describe any authentication procedures for each seed stock used or novel genotype generated. Describe any experiments used to assess the effect of a mutation and, where applicable, how potential secondary effects (e.g. second site T-DNA insertions, mosiacism, off-target gene editing) were examined.*
